# 固相萃取-气相色谱法测定橄榄油中4种脂肪酸乙酯

**DOI:** 10.3724/SP.J.1123.2022.09011

**Published:** 2023-04-08

**Authors:** Huiyuan LU, Lijuan WANG, Jiongkai ZHANG, Chizhong ZHANG, Tianjuan LI, Ruixue JI, Weijian SHEN

**Affiliations:** 1.南京海关动植物与食品检测中心, 江苏 南京 210001; 1. Animal, Plant, and Food Inspection Center of Nanjing Customs, Nanjing 210001, China; 2.上海安谱实验科技股份有限公司, 上海 201609; 2. Anpel Laboratory Technologies (Shanghai) Inc., Shanghai 201609, China

**Keywords:** 固相萃取, 气相色谱, 脂肪酸乙酯, 棕榈酸乙酯, 亚油酸乙酯, 油酸乙酯, 硬脂酸乙酯, 橄榄油, solid phase extraction (SPE), gas chromatography (GC), fatty acid ethyl esters (FAEEs), ethyl palmitate, ethyl linoleate, ethyl oleate, ethyl stearate, olive oil

## Abstract

橄榄油中脂肪酸乙酯(FAEEs)的含量是衡量其产品品质的重要指标之一,GB/T 23347-2021《 橄榄油、油橄榄果渣油》中要求特级初榨橄榄油中FAEEs应≤35 mg/kg。目前橄榄油中FAEEs的国际标准检测方法是硅胶柱层析-气相色谱法(GC),该方法存在操作复杂、耗时长、试剂消耗量大等缺点。本研究建立了硅胶固相萃取(Si SPE)-气相色谱分析橄榄油中棕榈酸乙酯、亚油酸乙酯、油酸乙酯和硬脂酸乙酯4种FAEEs的检测方法。考察了载气类型,选定He气作为载气;筛选了内标化合物,确定使用十七碳烯酸乙酯(顺-10)作为内标物质;优化了SPE条件,对比了不同品牌Si SPE柱对回收率的影响,确定了称取0.05 g橄榄油,使用正己烷提取,1 g/6 mL Si SPE柱净化的前处理方法。该方法处理一个样品大约仅需2 h前处理时间和23 mL试剂。对优化后的方法进行方法学验证,结果表明,4种FAEEs在0.1~5.0 mg/L的范围内线性关系良好(决定系数(*R*^2^)>0.999),方法检出限为0.78~1.11 mg/kg,定量限为2.35~3.33 mg/kg,在低、中、高3个加标水平(4、8、20 mg/kg)下的回收率为93.8%~104.0%,相对标准偏差为2.2%~7.6%。用建立的方法对15个橄榄油样品进行测试,发现3个特级初榨橄榄油样品的FAEEs总量超过了35 mg/kg,表明市场上可能存在以次充好的现象。与国际标准检测方法相比,该方法前处理过程简便,操作时间短,试剂用量少,检测成本低,精密度高,准确性好,可以为橄榄油检测标准的完善提供有效的理论和数据参考。

## Abstract (en)

The fatty acid ethyl ester (FAEE) content of olive oil is an important indicator of its quality. At present, the international standard method used to detect FAEEs in olive oil is silica gel (Si) column chromatography-gas chromatography (GC); however, this technique presents a number of disadvantages, including complex operation, long analysis times, and high reagent consumption. In this study, a method based on Si solid phase extraction (SPE)-GC was established to determine four FAEEs in olive oil, namely, ethyl palmitate, ethyl linoleate, ethyl oleate, and ethyl stearate. First, the effects of the carrier gas were investigated, and He gas was ultimately selected as the carrier gas. Next, several internal standards were screened, and ethyl heptadecenoate (*cis*-10) was determined as the optimal internal standard. The SPE conditions were also optimized, and the effects of different brands of Si SPE columns on the recoveries of analytes were compared. Finally, a pretreatment method in which 0.05 g of olive oil was extracted with *n*-hexane and purified through a Si SPE column (1 g/6 mL) was developed. A sample could be processed within approximately 2 h using a total reagent volume of about 23 mL. Validation of the optimized method revealed that the four FAEEs have good linearities within the range of 0.1-5.0 mg/L (coefficients of determination (*R*^2^)>0.999). The limits of detection (LODs) of the method were within 0.78-1.11 mg/kg, and its limits of quantification (LOQs) were in the range of 2.35-3.33 mg/kg. The recoveries ranged from 93.8% to 104.0% at all spiked levels tested (4, 8, and 20 mg/kg), and the relative standard deviations were 2.2%-7.6%. Fifteen olive oil samples were tested using the established method, and the total FAEEs of three extra-virgin olive oil samples were found to exceed 35 mg/kg. Compared with the international standard method, the proposed method has the advantages of simpler pretreatment process, shorter operation time, lower reagent consumption and detection cost, high precision, and good accuracy. The findings provide an effective theoretical and practical reference for improving olive oil detection standards.

初榨橄榄油是指用橄榄树的新鲜果实未经加热和化学处理,直接冷榨得到的油脂^[1]^。橄榄油营养成分丰富、保健功能突出,被公认为绿色保健食用油,具有预防心脑血管疾病、糖尿病、防癌、抗衰老等功能,在西方有“食用油皇后”“液体黄金”之美称^[2]^,因而越来越受广大消费者的喜爱。由于生产原料、加工工艺、杂质含量、感官评定等的不同,橄榄油被划分为不同的等级,其中特级初榨橄榄油是品质最好、质量控制最为严格的一种橄榄油,其价格也相对更高,因此市场上存在部分不良商家将初榨橄榄油充当特级初榨橄榄油来销售的现象。

受原料品质及保存条件等因素的影响,橄榄油中的脂肪酸与乙醇反应,易生成脂肪酸乙酯(fatty acid ethyl esters, FAEEs),从而影响橄榄油品质^[3]^,因此FAEEs含量是评价初榨橄榄油是否达到特级初榨橄榄油要求的重要指标之一。GB/T 23347-2021^[4]^和国际油橄榄理事会(International Olive Council, IOC)制定的标准COI/T.15/NC No 3/Rev.18/2022^[5]^规定了特级初榨橄榄油中FAEEs的总量不得超过35 mg/kg。GB/T 23347-2021^[4]^规定的FAEEs测试方法是2017年IOC发布的COI/T.20/Doc. No 28/Rev.2^[6]^,该方法采用自制的硅胶层析柱进行净化,气相色谱法(GC)测定,总体上存在前处理时间长、试剂消耗量大、工作效率低等缺点。

2019年,Uncu等^[7]^利用傅里叶变换红外吸收光谱技术(FTIR)和紫外-可见(UV-Vis)光谱技术分析了脂肪酸烷基酯,但该方法仅能检测总量,而无法精确测定单一化合物的含量。侯靖等^[3,8]^在2018年提出了用气相色谱-质谱联用法(GC-MS)检测橄榄油中脂肪酸烷基酯的方法,并于2020年进一步优化了检测方法,然而该方法已经滞后于IOC的最新规定(只需测定4种FAEEs),且杂质干扰及分离度问题仍然存在。基于此,本研究对IOC最新要求的测定橄榄油中4种FAEEs的前处理方法及仪器分析条件进行了针对性优化,并考察了方法的检出限、精密度和回收率等指标,以求为橄榄油中4种FAEEs的测定提供一种高效、准确、稳定和经济的检测方法。

## 1 实验部分

### 1.1 仪器、试剂与材料

Agilent 7890B气相色谱仪(美国Agilent公司),EOAA-HM-01多管漩涡混合器、DC系列24位防腐型水浴氮吹仪、CNWBOND硅胶(Si)固相萃取(SPE)柱(上海安谱实验科技股份有限公司); Agilent Si SPE柱(美国Agilent公司); Agela Cleanert Silica SPE柱(天津博纳艾杰尔科技有限公司); Waters Sep-Pak Vac Silica SPE柱(美国Waters公司); Supelco LC-Si SPE柱(美国Supelco公司), 5个品牌的硅胶SPE柱的填料均为裸硅胶,未经任何官能团修饰。

正己烷、正庚烷、乙醚均为色谱纯,棕榈酸乙酯(C_16∶0_E)、亚油酸乙酯(C_18∶2_E)、油酸乙酯(C_18∶1_E)、硬脂酸乙酯(C_18∶0_E)、十七烷酸甲酯(C_17∶0_M)、十七烷酸乙酯(C_17∶0_E)、十七碳烯酸乙酯(顺-10)(C_17∶1_E)纯度均大于99%,购自上海安谱实验科技股份有限公司。橄榄油样品购自本地超市。

### 1.2 标准溶液的配制

准确称取FAEEs标准物质各10 mg(精确到0.01 mg)于10 mL容量瓶中,用正庚烷溶解并定容,配制成1.0 g/L单标储备溶液。将棕榈酸乙酯、亚油酸乙酯、油酸乙酯、硬脂酸乙酯4种FAEEs单标储备溶液用正庚烷稀释成50 mg/L的混合外标中间溶液。将C_17∶1_E的单标储备溶液用正庚烷稀释成50 mg/L的内标中间溶液。-18 ℃避光保存于玻璃瓶中,有效期6个月。

取适量混合外标中间溶液和内标中间溶液用正己烷稀释成外标质量浓度分别为0.1、0.5、1、2和5 mg/L、内标质量浓度均为2 mg/L的系列混合标准工作液,现配现用。

### 1.3 实验条件

#### 1.3.1 样品前处理

提取:称取0.05 g(精确至0.0001 g)橄榄油样品于10 mL玻璃试管中,加内标中间溶液80 μL,混匀,再加入正己烷2 mL,涡旋混匀30 s,待净化。

净化:Si SPE柱用正己烷6 mL活化,将待净化液转移至活化后的Si SPE柱,自然滴下,待样液过柱后,加入15 mL的正己烷-乙醚溶液(99∶1, v/v)进行洗脱,弃去前5 mL流出液,收集后10 mL的洗脱液,待浓缩。

浓缩:于40 ℃氮吹至近干,加入2 mL正己烷涡旋溶解后,过0.45 μm有机相滤膜,滤液待测。

#### 1.3.2 气相色谱条件

DB-5毛细管色谱柱(30 m×0.25 mm×0.25 μm);柱升温程序:初始温度50 ℃,以30 ℃/min升至100 ℃保持1 min,以20 ℃/min升至200 ℃,以5 ℃/min升至240 ℃,以35 ℃/min升至310 ℃保持3 min;载气为高纯He气(纯度≥99.999%);恒流模式,流速1.0 mL/min;进样口温度300 ℃;进样量1.0 μL;不分流进样,1.5 min后打开分流阀;FID检测器温度300 ℃。

## 2 结果与讨论

### 2.1 方法优化

#### 2.1.1 载气的确认

载气是影响气相色谱柱分离效能和检测器灵敏度等的重要因素^[9,10]^。本文考察了N_2_(纯度≥99.999%)和He两种载气条件下FAEEs的分离情况(见图1)。结果发现:使用不同载气,4种FAEEs的保留时间和分离度均有一定差异。其中,C_16∶0_E、C_18∶0_E、C_17∶1_E之间的分离度均较好,大于1.5,但C_18∶1_E和C_18∶2_E在较短的分析时间内较难分离,N_2_或He作载气时分离度分别为0.94和1.24,因此最终确定以He作为载气。此结果与裴紫薇等^[11]^报道的He对顺反式脂肪酸异构体的分离和准确定量均优于N_2_的结果一致。

**图1:**
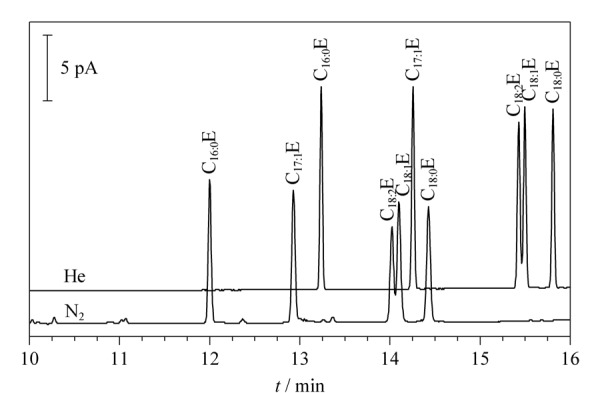
不同载气条件下FAEEs标准溶液的色谱图

#### 2.1.2 内标物质选择

为了尽可能减少前处理操作引起的误差,并提高仪器分析时进样重复性和定量精准度,本方法采用内标法进行定量分析。根据目标物的理化性质,拟选择结构性质相似的C_17∶0_E和C_17∶1_E作为备选内标,考察其与目标物的分离情况,以及基质干扰情况,以筛选出合适的内标物质。结果如图2所示,两种内标与4种FAEEs分离度均较好,但C_17∶0_E和基质中杂质峰保留时间存在部分重叠,而C_17∶1_E不受杂峰干扰,故最终选择C_17∶1_E作为内标物质。

**图2:**
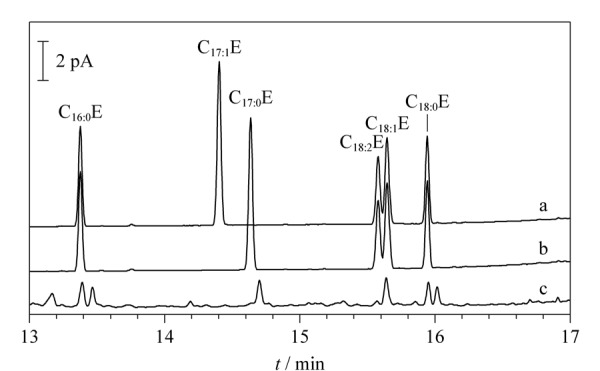
以(a) C_17∶1_E或(b) C_17∶0_E作内标的FAEEs标准溶液以及(c)无内标的样品溶液的色谱图

#### 2.1.3 SPE方法优化

近年来,硅胶作为吸附填料被普遍应用于脂肪酸烷基酯类化合物的分离纯化过程^[12⇓-14]^。

Valli等^[15]^发现Si SPE柱填料变异性对实验的影响较手动装填的长硅胶玻璃柱更小,具有良好的重复性。因此,本文采用CNW Si SPE柱作为橄榄油中FAEEs检测的净化柱,并对Si SPE柱的柱规格及油样的称样质量进行优化,实验步骤参照1.3.1节进行,待提取液过柱后,用正己烷-乙醚(99∶1)溶液进行洗脱,并分步收集每毫升洗脱液进行测试,研究不同条件下FAEEs在Si SPE柱上的洗脱规律,本研究共在6种方案下进行洗脱测试,具体方案情况和相应目标物洗脱结果如图3所示。

**图3:**
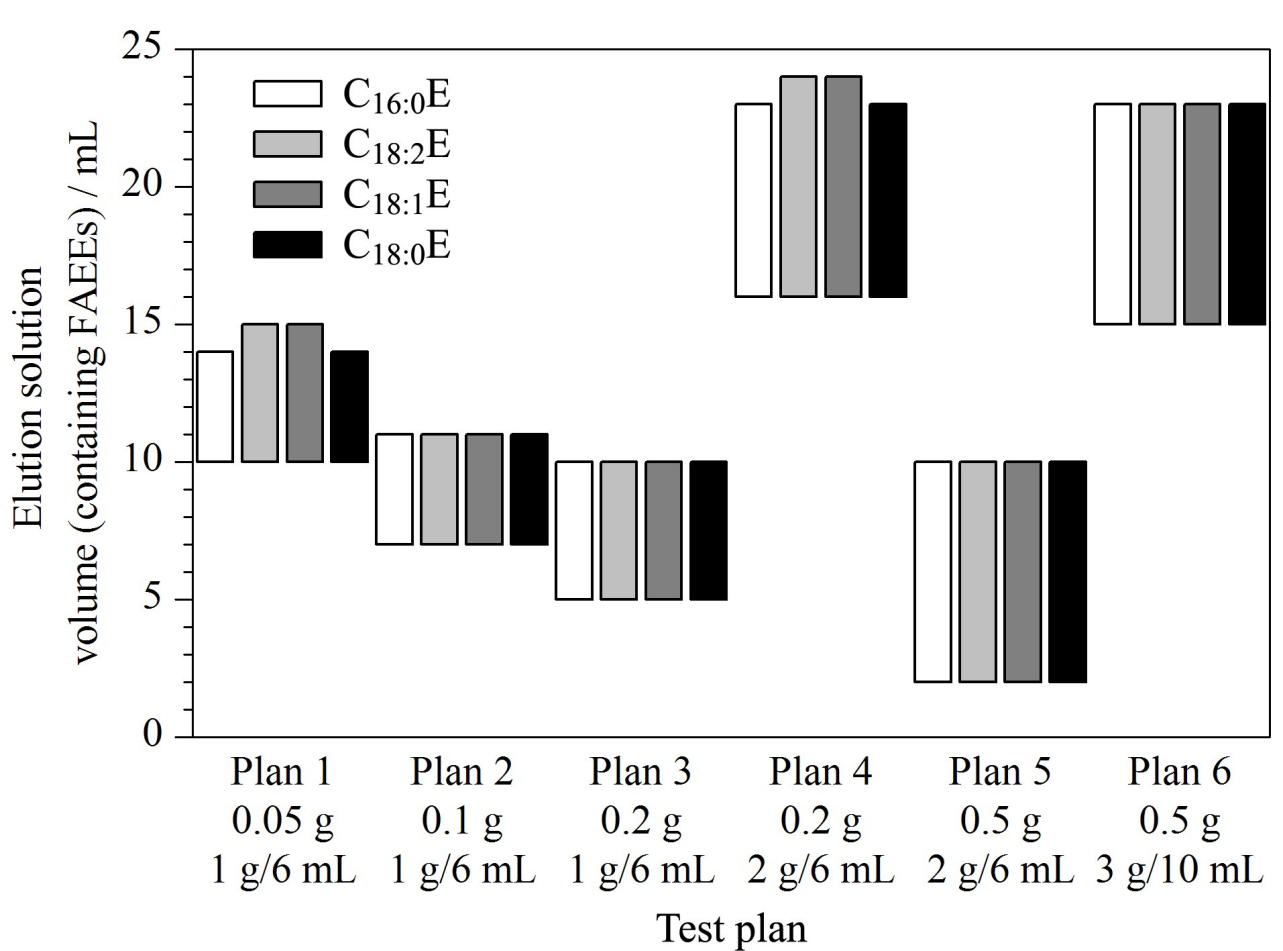
采用不同洗脱方案时FAEEs的洗脱结果

结果显示:SPE柱填料质量相同时,目标物开始流出的时间会随样品质量增加而提前,目标物全部流出所消耗的洗脱液体积会相应减少;当样品质量相同时,SPE柱填料质量增加,目标物全部流出所消耗的洗脱液体积增大,应该是目标物在SPE柱内的保留加强导致流出较晚。即Si SPE柱的柱规格和油样质量,均会影响SPE柱对目标化合物的吸附和解吸附能力。

此外,在洗脱液合并浓缩时发现:方案3中个别样品存在微量油滴,方案5和方案6所有样品均有明显油滴。此现象是样品过载导致的。Si SPE的净化效果不理想,油脂等杂质成分未去除,如直接进样分析,会对仪器的进样口、色谱柱、检测器造成严重的污染,还会影响测定结果的准确性。综合考虑实验效率、实验成本以及净化效果等因素,最终选择方案1:用1 g/6 mL Si SPE柱对0.05 g橄榄油样品进行净化,总洗脱液体积为15 mL。

#### 2.1.4 不同品牌Si SPE柱普适性考察

由于不同品牌的Si SPE柱的吸附净化能力受填料、装填工艺等因素的影响,为评估不同品牌的Si SPE柱对本前处理方法的适用性,本文选择了市场上5个国内外品牌的Si SPE柱(1 g/6 mL)进行验证。实验步骤参照1.3.1节进行,待提取液过柱后,用正己烷-乙醚(99∶1)溶液进行洗脱,并分步收集第1~18 mL的洗脱液进行测试。使用Si SPE柱净化时橄榄油中FAEEs洗脱曲线图见图4(以CNW和Supelco为例),不同品牌的Si SPE柱中FAEEs的流出趋势相似,均呈现出目标物浓度先显著增加,后逐步降低的现象,但不同品牌的小柱目标物集中流出时间稍有差异。如采用CNW Si SPE柱净化时,目标物集中在第11~15 mL之间流出,而使用Supelco Si SPE柱净化时,目标物集中在第7~10 mL之间流出,Agilent、Agela、Waters 3个品牌的Si SPE柱与CNW Si SPE柱相对接近,均在第11~15 mL之间流出。考虑到使用不同品牌的Si SPE柱净化时,目标物流出情况的差异,为了避免目标物损失,同时尽可能减少洗脱液接收量以提高氮吹浓缩效率,本文选择用15 mL正己烷-乙醚混合液(99∶1)进行洗脱,弃去前5 mL的洗脱液,收集后续流出的10 mL洗脱液进行浓缩、复溶和检测。

**图4:**
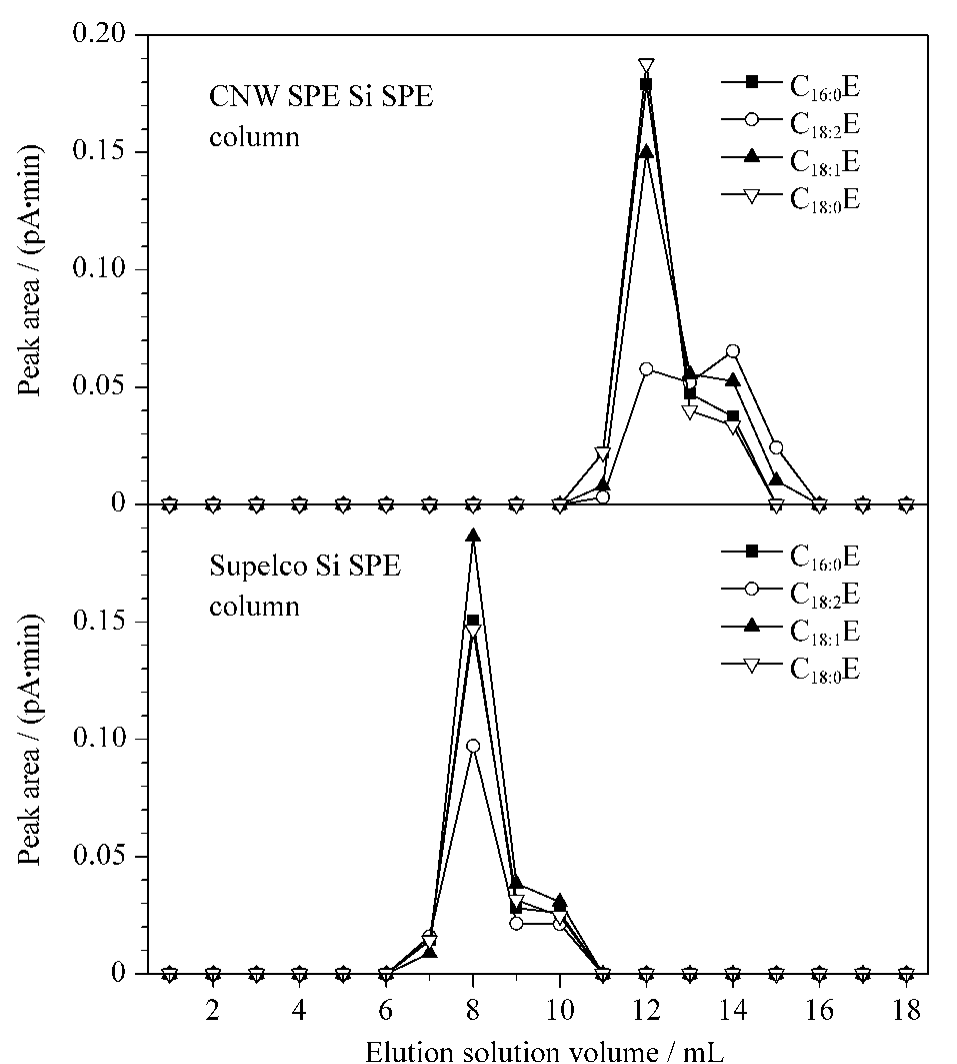
采用不同品牌的Si SPE柱净化时FAEEs的洗脱曲线图

为进一步验证不同品牌Si SPE柱对橄榄油中4种FAEEs检测准确性的影响。选择某一品牌特级初榨橄榄油样品,进行加标试验,考察加标回收率情况,结果如图5所示,用不同品牌的Si SPE柱净化后,橄榄油中4种FAEEs的回收率良好,无明显差异,均在94.2%~106.2%之间,表明该前处理方法对不同品牌的Si SPE柱均适用。

**图5:**
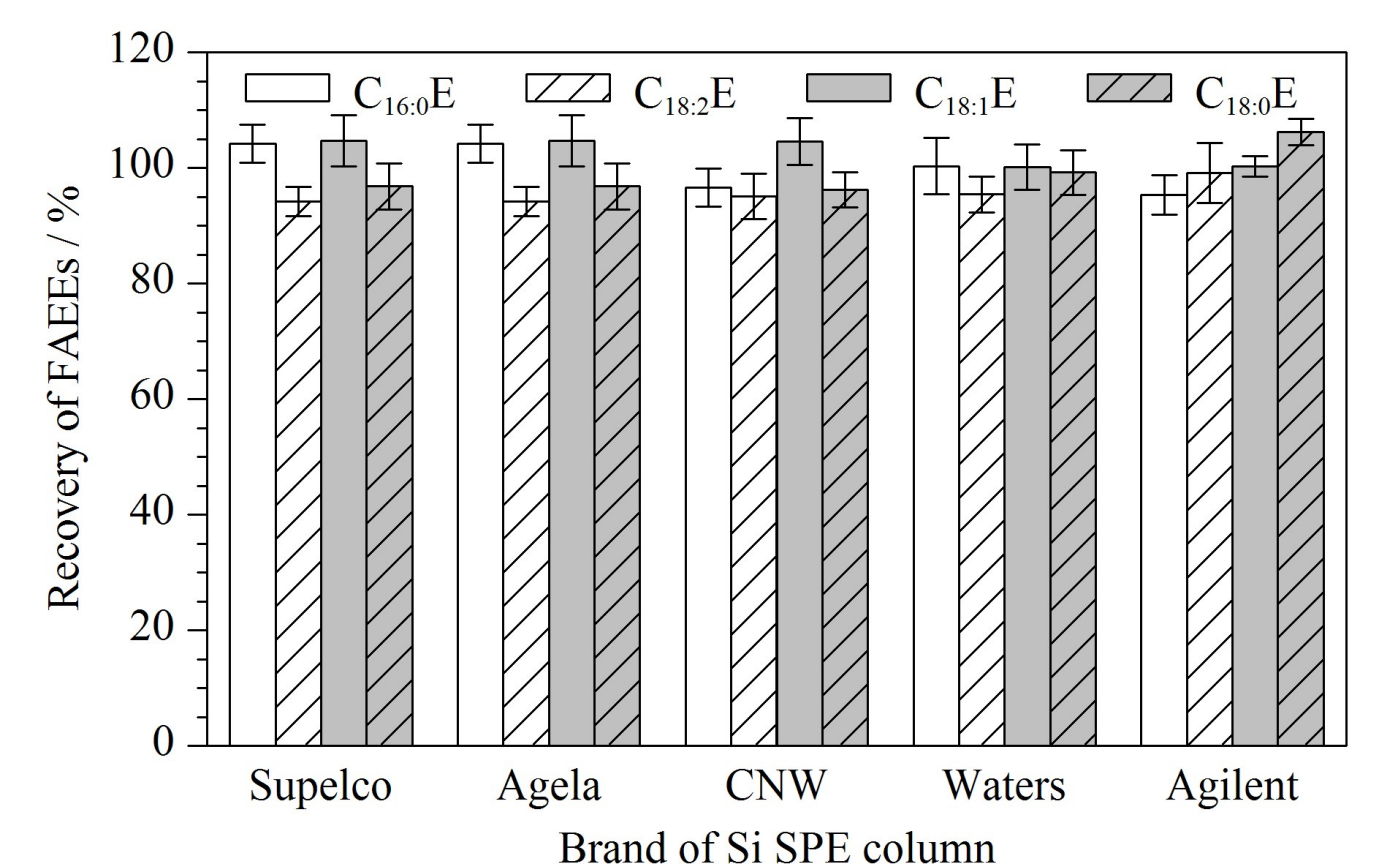
不同品牌的硅胶固相萃取柱净化后FAEEs的回收率(*n*=3)

#### 2.1.5 与IOC方法比较

分别采用本方法与IOC的方法(COI/T.20/Doc. No 28/Rev.2)对橄榄油中FAEEs进行检测,气相色谱图见图6,加标回收率结果见图7。结果显示,与IOC的方法比较,虽然两种方法加标回收率均在90%~110%之间,但本方法的基线相对更稳定,基本无干扰,且采用本方法处理一个样品耗时仅约2 h,试剂消耗约23 mL,而IOC方法耗时20 h以上,试剂消耗550 mL以上。综上所述,本方法在简化前处理步骤、降低实验成本、保证结果准确性和对环境、人员友好性方面均有明显的优势。

**图6:**
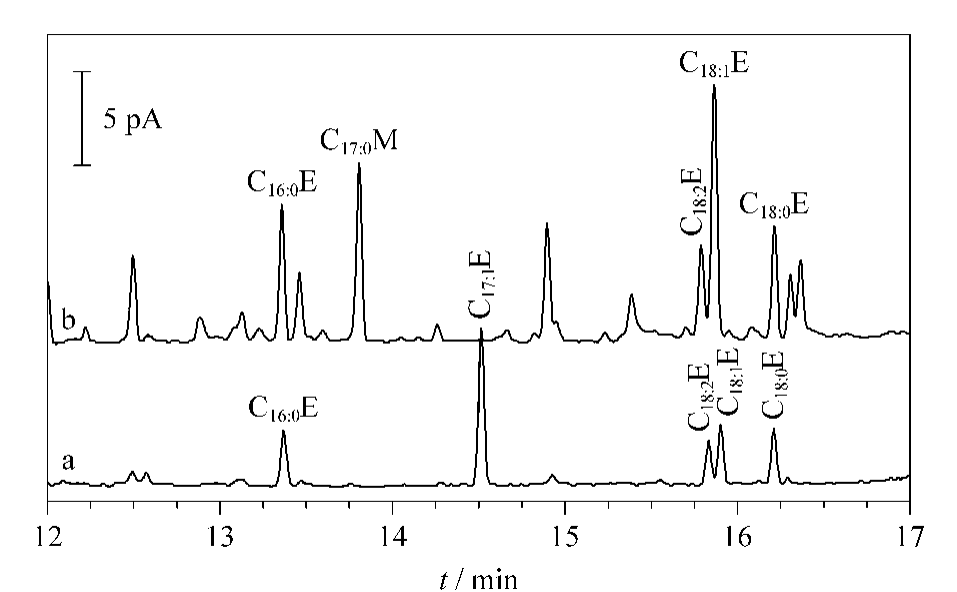
两种前处理方法所得的样品色谱图

**图7:**
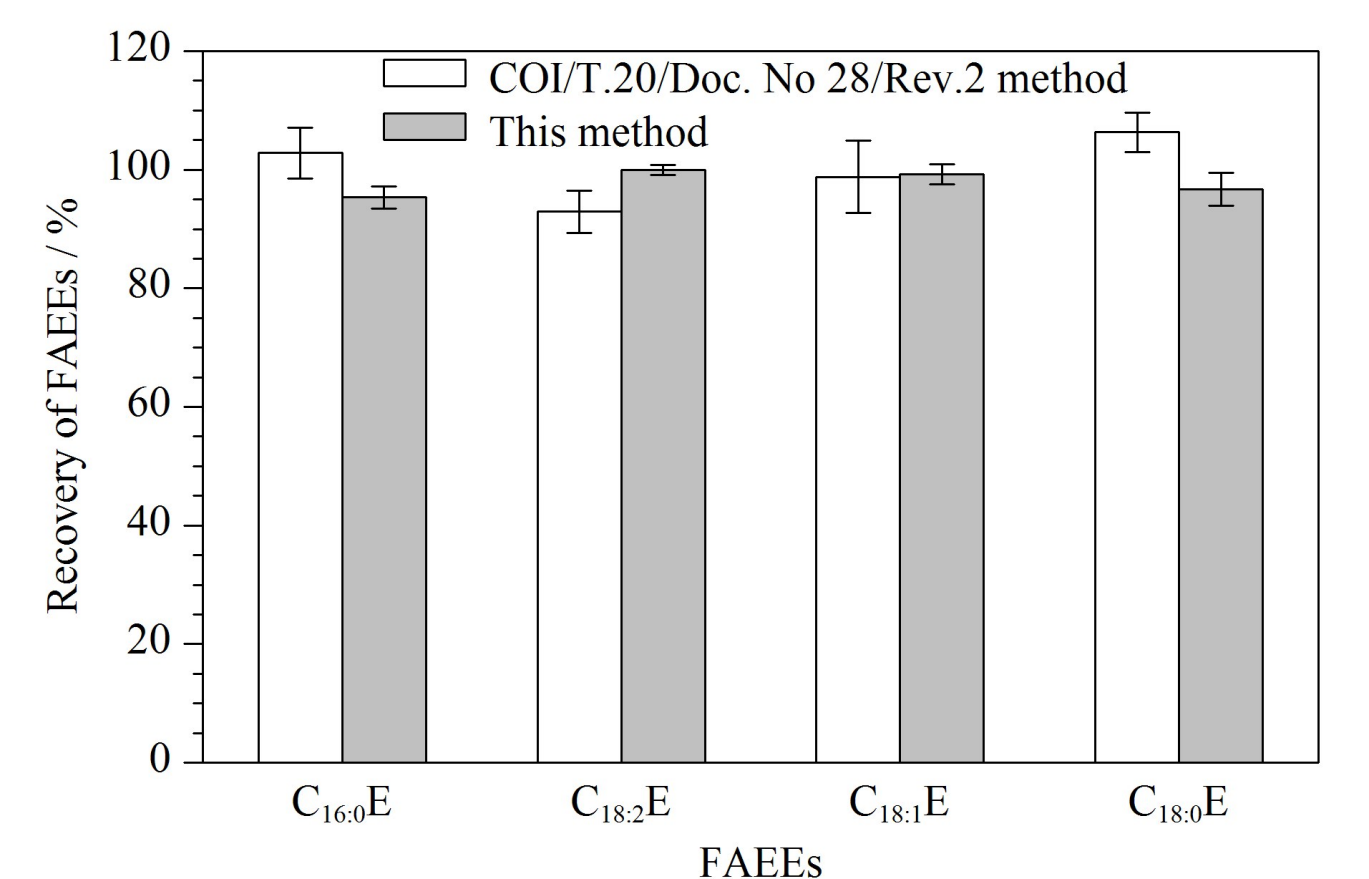
两种前处理条件下样品的加标回收率(4 mg/kg)

### 2.2 方法学考察

#### 2.2.1 线性范围、检出限及定量限

以C_17∶1_E为内标,目标物的质量浓度为横坐标*x*,峰面积比为纵坐标*y*绘制标准曲线,结果显示,4种FAEEs在0.1~5.0 mg/L内线性良好,见表1。

**表1:** FAEEs的回归方程、决定系数(*R*^2^)、检出限和定量限

Compound	Regression equation	R^2^	LOD/ (mg/kg)	LOQ/ (mg/kg)
C_16∶0_E	y=0.5051x-0.0104	0.9999	0.78	2.35
C_18∶2_E	y=0.3806x-0.0099	0.9999	0.85	2.56
C_18∶1_E	y=0.4944x-0.0074	0.9999	1.11	3.33
C_18∶0_E	y=0.4825x-0.0085	0.9999	0.82	2.45

*y*: peak area ratio; *x*: mass concentration, mg/L.

参考GB/T 27417-2017^[16]^,采用空白标准偏差法评估方法的检出限(LOD),对低浓度样品独立测试10次,分别以结果的3倍标准偏差作为方法检出限,以3倍方法检出限作为方法的定量限(LOQ)。结果见表1,显示4种FAEEs的方法LOD和LOQ分别为0.78~1.11 mg/kg和2.35~3.33 mg/kg。

#### 2.2.2 回收率和精密度

根据特级初榨橄榄油中FAEEs的限值(总和不超过35 mg/kg)^[4,5]^和GB/T 27404-2008^[17]^对限量指标的回收率测试要求,设定3个加标水平:4、8、20 mg/kg(分别约相当于4种FAEEs总和的定量限、限量指标、限量指标的2倍),每个水平平行检测6次,分别计算回收率和精密度,结果见表2。由表2数据可以看出,FAEEs的回收率均在93.8%~104.0%之间,RSD均≤7.6%,表明本方法准确度和精密度良好。

**表2:** FAEEs在橄榄油中3个水平下的平均回收率及相对标准偏差(*n*=6)

Compound	Background/ (mg/kg)	Added/ (mg/kg)	AR/ %	RSD/ %
C_16∶0_E	3.25	4	95.3	4.3
		8	99.8	6.0
		20	104.0	2.2
C_18∶2_E	1.68	4	100.0	6.5
		8	102.8	2.3
		20	100.2	4.3
C_18∶1_E	3.78	4	99.3	6.1
		8	93.8	3.6
		20	102.7	2.8
C_18∶0_E	3.79	4	96.7	3.3
		8	100.3	7.6
		20	100.1	4.2

### 2.3 实际样品检测

在超市随机选购15个不同产地、不同品牌和等级的橄榄油样品,对本方法的普适性进行验证。样品信息及测试结果见表3。测试的15个橄榄油样品中有12个特级初榨橄榄油、3个精炼和特级初榨混合橄榄油,结果显示3个特级初榨橄榄油样品的FAEEs总量超过35 mg/kg。实验结果表明市场上可能存在以次充好的现象。

**表3:** 实际橄榄油样品中FAEEs的测试结果

No.	Grade	Origin	Contents/(mg/kg)				
			C_16∶0_E	C_18∶2_E	C_18∶1_E	C_18∶0_E	SUM
1	refined+extra virgin olive oil	Italy	N. D	16.09	7.47	N. D	23.56
2	extra virgin olive oil	Spain	2.48	3.41	3.16	N. D	9.05
3	extra virgin olive oil	Spain	3.09	13.83	10.74	N. D	27.66
4	extra virgin olive oil	Spain	3.25	1.68	3.78	3.79	12.5
5	extra virgin olive oil	Spain	3.31	7.76	7.86	N. D	18.93
6	extra virgin olive oil	Spain	3.87	7.89	20.57	N. D	32.33
7	extra virgin olive oil	Greece	8.08	7.13	41.54	3.17	59.92
8	extra virgin olive oil	Italy	N. D	5.79	13.37	N. D	19.16
9	extra virgin olive oil	Spain	4.43	8.99	21.27	N. D	34.69
10	Refined+extra virgin olive oil	Spain	4.02	12.32	46.14	N. D	62.48
11	extra virgin olive oil	Spain	14.78	39.2	195.01	8.56	257.55
12	extra virgin olive oil	Portugal	3.36	6.15	9.98	N. D	19.49
13	extra virgin olive oil	Spain	6.18	5.25	18.33	3.12	32.88
14	extra virgin olive oil	Italy	4.95	20.48	28.52	N. D	53.96
15	refined+extra virgin olive oil	Italy	2.87	16.79	14.66	N. D	34.32

N. D.: not detected (below the LOQ).

## 3 结论

本文建立了采用Si SPE-GC检测橄榄油中4种FAEEs的方法,经过优化和验证,建立的方法定性可靠、定量准确,前处理简单、高效、经济、环保,与现行有效的国际标准相比,具有一定的优越性和可替代性,可以为规范橄榄油市场提供技术支撑。
